# Employment of Electricity and Electro-Magnetism in Poisoning by Laudanum

**Published:** 1844-06

**Authors:** George Corfe


### SELECTIONS

FROM

### AMERICAN AND FOREIGN JOURNALS.

*Employment of Electricity and Electro-Magnetism in Poisoning by Laudanum.* By George Corfe, Esq. Sir—I beg leave to submit to you the following particulars relating to two persons who were brought into the hospital under the denomination of "poison cases;" the first of which was really one, the second had all the appearance of being so, both from the symptoms that presented themselves and from strong circumstantial evidence.

Both cases were treated alike; the first recovered perfectly, the latter one died; and it was by means of the post-mortem examination that the real cause of her decease was discovered to be in that change of structure in the kidneys known as "Bright's disease."

With reference to the former I will observe, that a square-built, stout man, (G. Freegroove), aged 54, was brought here at three o'clock in the morning of the 14th of September last, having taken an ounce and a half of laudanum the preceding evening, at half past nine o'clock. In the first instance I ordered the administration of the stomach-pump, at which period, to all appearances, he was a lifeless corpse: the pupils were contracted to a pin-hole in size; the pulse was intermitting, and not more than 40; the respirations convulsively performed at intervals of half a minute; the face livid, and the extremities bluish and cold. After the stomach had been relieved of its contents, green tea, with ammonia was injected therein; flaggellation with thin splints and wet towels, the cold douche, turpentine stupes and sinapisms to his calves and abdomen, were applied in succession, without the least improvement in his condition. The bladder was relieved of six or eight ounces of light-colored urine by the catheter. I then thought of a most powerful remedy, which was attended with extraordinary success; I allude to the electro-magnetic battery, conjointly with electricity, which was set to work upon him soon after four o'clock. The pulse became more steady, firm, and frequent; the respiration more indicative of resuscitation. Our powerful electrical machine was now got into full play before a large fire, and the jar filled, when some brilliant sparks and strong shocks were occasionally passed through his head, spine, thorax, and abdomen. None of these means roused him, beyond throwing the whole body into violent and convulsive succussions and muscular contractions.

After an hour's hard work, in which the son of the patient exerted himself most indefatigably, the man suddenly roused up, with something like the following exclamation:—"Holloa! you rascals, what are you trying specimens on me for? I wish you'd let me alone." At this moment he opened his eyes and looked wildly about him, and though he evidently did not recognize the person of his son, yet he knew his voice, and asked him for his clothes to put on, which he said were in the next room, whilst the poor son was so affected by the sudden change from apparent death to comparative consciousness, that he turned aside and gave expression to his feelings by bursting into tears. After this the patient relapsed a little, and another shock was given him from time to time, which, when it was directed to the tip of the nose, excited his frame and his anger too, above all spots, for he instantly broke out in such remonstrances as these:—"What are you about, you set of beasts! You are only keeping me here to try your specimens upon me. I want to go, and you shan't keep me either, you unfeeling brutes! You are like savages, pulling a man about in this way," &c.

This work went on until after seven o'clock, when I considered him so much better as to place him in the ward in charge of the nurse and his son, and he walked home from the hospital the following day, quite well and very sulky.

I now proceed to the second case, that of a corpulent widow, about 40 years of age, from Rathbone-place, who was brought here by the police on the 5th of December, at 4 o'clock, p. m., under very similar circumstances, being found in her bed at six o'clock, a. m., in a state of perfect insensibility, which had continued ever since. The female, her companion, who slept in the next room, stated that she had taken, in the course of the night, about three ounces of laudanum. The pulse, however, was slow, but regular; the respirations irregular and convulsively performed, about six and eight in a minute; the pupils were contracted, but not so much so as in the previous case. The treatment was precisely the same, except that we could not succeed in emptying the stomach, though we injected green tea and ammonia by means of a large catheter and an elastic gum bottle. She died four hours and a half after her admission, or fifteen hours from the period when she was discovered to be in this state.

On informing you, sir, of this case, I received your order for examining the body, which I accordingly did about 18 hours after death. The left hemisphere of the brain was flatter and not so fully developed as the right, otherwise there was no structural disease or lesion of this organ. The contents of the thorax were healthy, the lungs gorged with black blood, and loose coagula of black blood in both ventricles of the heart. The mucous membrane of the stomach was softened, and easily torn away by the nail; it contained only the green tea which had been injected.

Having made the examination, I came to the conclusion that the deceased had not destroyed herself by laudanum. I left the body, and it was sewed up for the coroner and jury, when, having pondered the case over, the conviction was brought to my mind that in the kidneys might be discovered the real cause of the lady's death. I lost no time, therefore, in getting the body re-opened, and in removing the kidneys, when, to my astonishment, and, I may say, great satisfaction, I found them to present a striking specimen of that disease known as "Bright's kidney," in its second stage; they emitted that peculiar rank, suffocating smell, not unlike game in a state of putrefaction, and which I frequently noticed before in cases of poison from "ischuria renalis." The bladder was firmly contracted and not larger than a walnut, and was even smaller than the uterus. This fact, I may observe, I noticed on the first day's examination.

It was now clear to my mind that the deceased had died from renal apoplexy, or as authors term it, ischuria renalis, a disease which has been so ably described by Sir Henry Halford, in a paper read before the Royal College of Physicians, and quoted by Dr. Watson, in his recently published and valuable lectures on the practice of physic, from which work I shall take the liberty of quoting a case exactly corresponding to that of the deceased.

"A very corpulent robust farmer, of about 55 years of age, was siezed with a rigor, which induced him to send for his apothecary. He had not made water, it appeared, for twenty-four hours. But there was no pain, no sense of weight in the loins, no distention of any part of the abdomen; and, therefore, no alarm was taken till the following morning, when it was thought proper to ascertain whether there was any water in the bladder, by the introduction of the catheter, and none was found. I was then called (says Sir Henry), and another inquiry was made, some few hours afterwards by one of the most experienced surgeons in London, whether the bladder contained any urine or not, when it appeared clearly that there was none. The patient sat up in bed and conversed as usual, complaining of some nausea, but of nothing material in his own view; and I remember that his friends expressed their surprise that so much importance should be attached to so little apparent illness. The patient's pulse was somewhat slower than usual, and sometimes he was heavy and oppressed. I ventured to state (continues the author) that if we should not succeed in making the kidneys act, the patient would soon become comatose, and would probably die the following night, for this was the course of the malady in every other case that I had seen. It happened so; he died in thirty hours after this, in a state of stupefaction."

Before I conclude, I must mention, as a fact of the case worthy of notice, that the shocks of electricity produced a faltering of the pulse and diminution of the vital powers, instead of rallying them, as was the case with the man.

I would leave these hints regarding this powerful therapeutical agent for your consideration, and that of any medical gentleman who may be connected with the Royal Humane Society, or other similar institutions.

I beg leave also to add, that during my residence in this hospital I have received several dozens of patients who had fallen down in what were called fits of insensibility, or apoplexy, but in all of which the same appearances which I have just described were visible after death; and in this instance my mind was strongly carried away from the consideration of such a disease existing by reason of the positive assertions of her friends that she had taken laudanum; whereas it appeared on the inquest that this drug, which was kept in several bottles by her, was only used as a liniment on her loins, to relieve the severe pains she felt there and in her head, for which cause she often said to her niece, who slept with her, that she should one day certainly go mad. It was fully proved by the niece that she had never taken laudanum, though the pain had been unusually great on the night (December 4) of her last attack of headache. These facts will, I think, appear worthy of attention.

*London Lancet*, for January.

